# Blood Vessels: The Pathway Used by Schwann Cells to Colonize Nerve Conduits

**DOI:** 10.3390/ijms23042254

**Published:** 2022-02-18

**Authors:** Benedetta Elena Fornasari, Federica Zen, Giulia Nato, Marco Fogli, Federico Luzzati, Giulia Ronchi, Stefania Raimondo, Giovanna Gambarotta

**Affiliations:** 1 Department of Clinical and Biological Sciences (DSCB), University of Torino, Regione Gonzole 10, 10043 Orbassano, TO, Italy; benedettaelena.fornasari@unito.it (B.E.F.); federica.zen@unito.it (F.Z.); giulia.ronchi@unito.it (G.R.); stefania.raimondo@unito.it (S.R.); 2 Neuroscience Institute Cavalieri Ottolenghi (NICO), University of Torino, 10043 Orbassano, TO, Italy; giulia.nato@unito.it (G.N.); marco.fogli@unito.it (M.F.); federico.luzzati@unito.it (F.L.); 3 Department of Life Sciences and Systems Biology (DBIOS), University of Torino, 10123 Torino, TO, Italy

**Keywords:** nerve guidance conduit, vascularization, repair Schwann cells

## Abstract

The repair of severe nerve injuries requires an autograft or conduit to bridge the gap and avoid axon dispersion. Several conduits are used routinely, but their effectiveness is comparable to that of an autograft only for short gaps. Understanding nerve regeneration within short conduits could help improve their efficacy for longer gaps. Since Schwann cells are known to migrate on endothelial cells to colonize the “nerve bridge”, the new tissue spontaneously forming to connect the injured nerve stumps, here we aimed to investigate whether this migratory mechanism drives Schwann cells to also proceed within the nerve conduits used to repair large nerve gaps. Injured median nerves of adult female rats were repaired with 10 mm chitosan conduits and the regenerated nerves within conduits were analyzed at different time points using confocal imaging of sequential thick sections. Our data showed that the endothelial cells formed a dense capillary network used by Schwann cells to migrate from the two nerve stumps into the conduit. We concluded that angiogenesis played a key role in the nerve conduits, not only by supporting cell survival but also by providing a pathway for the migration of newly formed Schwann cells.

## 1. Introduction

Traumatic lesions of the peripheral nerves are a major health problem characterized by a high incidence in the population and their consequences could lead to long-lasting disability and functional deficits [1].

The intrinsic regenerative capacity of the peripheral nervous system is not sufficient to guarantee a satisfactory regeneration after severe damage; in fact, all nerve injuries with loss of a nerve portion are classified as “severe nerve injuries” but, depending on the gap length, the outcome of regeneration may be different, with a greater chance of success in the case of a small gap repair [2,3].

When the gap is a few millimeters, the formation of a 3D structure characterized by a matrix, fibroblasts, perineurial, and inflammatory cells called the “nerve bridge” between the proximal and the distal stump was observed [4,5,6]. Within this 3D structure, macrophages sensing a hypoxic condition produce and secrete vascular endothelial growth factor-A (VEGF-A), which, through a chemotactic signal, guides endothelial cell proliferation and vessel formation [6]. Intriguingly, blood vessels were shown to provide a track for Schwann cell migration [6], a process that is necessary for axonal pathfinding across the nerve bridge [7]. In general, Schwann cell migration is not only necessary for axonal pathfinding in the nerve bridge, but it is one of the intrinsic steps required for nerve regeneration for all nerve injuries, regardless of the type of damage and repair [8,9]. Different factors might be involved in Schwann cell migration and proliferation after an injury, among them, the soluble isoform of Neuregulin 1 (NRG1) [10], a factor expressed not only by Schwann cells, but also by rat and human nerve fibroblasts [11,12], which also express growth arrest-specific gene 6 (GAS6), a mitogenic factor for Schwann cells [13], and fibroblast growth factor 5 (FGF5) [12], a factor regulating Schwann cell adhesion and migration [14].

While many details have been disclosed about nerve regeneration in the nerve bridge [15], little is known about the possible interaction between Schwann cells and endothelial cells during regeneration within a conduit used to repair a severe nerve injury with a longer gap.

Nerve regeneration within a conduit is significantly different from what occurs within a nerve bridge. Indeed, the nerve bridge does not require repair since it is a tissue that spontaneously forms between adjacent injured stumps, while to bridge the gap between the proximal and the distal stump when the gap is large, a conduit is required [16]. Moreover, the conduit environment is enclosed, while the regenerating nerve bridge is in direct contact, and possibly affected, by the surrounding tissue. Conduits in place of the autografts (the gold standard technique) allow for overcoming the side-effects caused by autologous grafting, such as a secondary surgery and sensitivity loss [8], and provide a protected environment and physical guidance for axonal growth [17,18,19].

Nevertheless, it must be considered that the environment within a conduit is completely different from that within the autograft; indeed, the conduit must be colonized by different cell populations, while, in the autograft, most of the actors involved in the nerve regeneration are already on site.

The spatiotemporal sequence of cellular events that characterize silicone conduit colonization was described several years ago through conventional light and electron microscopy analyses. Conduits are first bridged by a mesh of fibrin deposits containing many erythrocytes, neutrophils, platelets, and macrophages [20,21]. Perineurial cells are the first cell type from both nerve stumps colonizing the conduit. After initial perineurial tube formation, nerve fibroblasts, endothelial cells, and Schwann cells [21,22] migrate in the conduits, followed by axon growth and myelination [20,21,22,23]. While these studies reported only qualitative observations, Li and colleagues [24] further quantified the percentage of different cell types that colonize a silicone conduit at different time points using electron microscopy, showing a large presence of capillaries; however, only general information on the interactions between the different cell types was reported.

Despite these previous studies, some aspects of nerve regeneration remain unclear, including how Schwann cells might migrate inside a conduit.

In a previous study [11], we detected an elevated presence of blood vessels inside the chitosan conduit at 7 days after repair, in contrast with the autograft, where blood vessels did not fill the autologous graft. This observation led us to investigate whether Schwann cells could use blood vessels inside the chitosan conduit as a track for their migration, as occurs in the nerve bridge [6].

## 2. Results

### 2.1. Migrating Schwann Cells Use Newly Formed Blood Vessels as a Substrate for Migration within the Conduit

**Figure 1:**
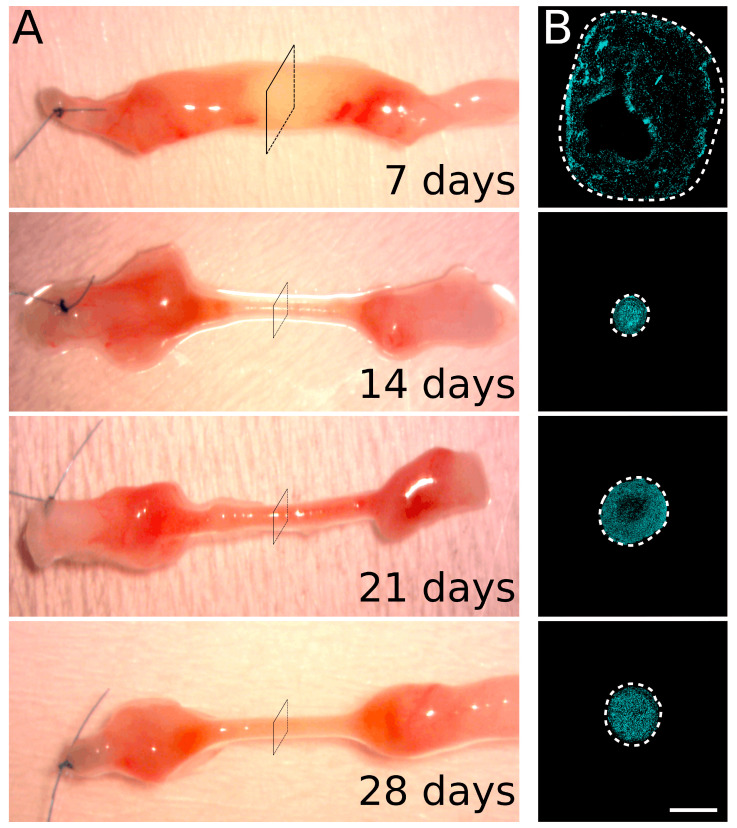
Sample collection. (**A**) Macroscopic representative images of regenerating nerves collected 7, 14, 21, and 28 days after the injury and repair. The chitosan conduit was removed and a single stitch identified the proximal stump of the nerve. (**B**) A representative section in the center of the conduit (corresponding approximately to the square drawn in panel **A**) labeled with the nuclear marker (DAPI, cyan). The dotted line delimits the region containing cell nuclei. Scale bar: 400 µm.

**Figure 2:**
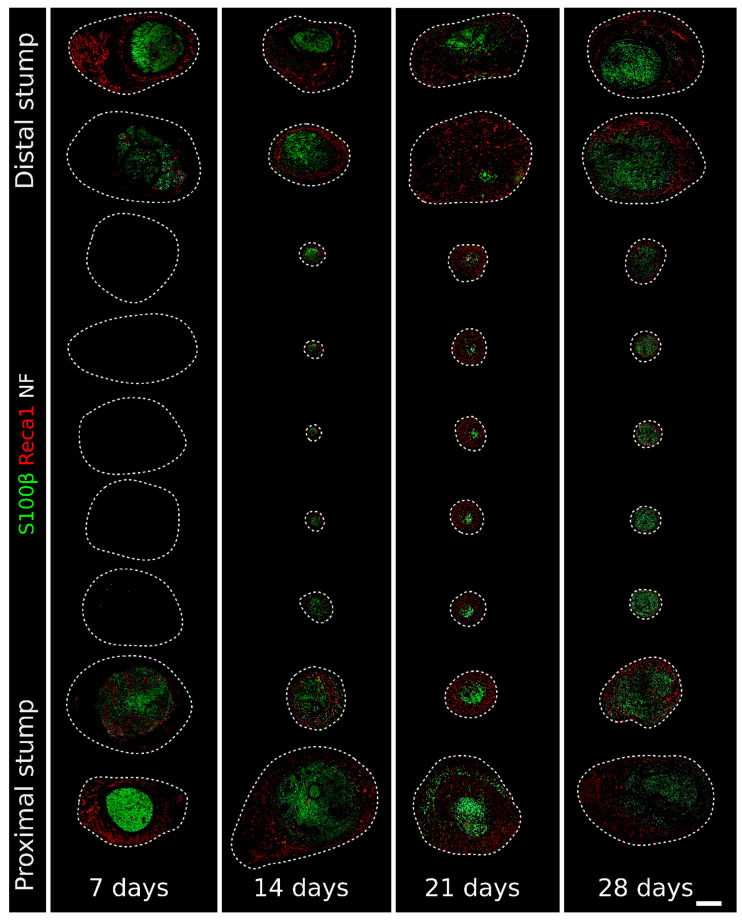
Immunofluorescence staining of regenerating nerves 7, 14, 21, and 28 days after the injury and repair. One section every millimeter labeled with Reca1 (red, endothelial cell marker), S100β (green, Schwann cell marker), and Neurofilament/NF (white, axon marker) to follow the nerve regeneration progression. The single labeling is shown in Figures S1 (NF), S2 (S100β), and S3 (Reca1). The dotted line delimits the region containing cell nuclei identified with DAPI (as in Figure 1B). Scale bar: 400 µm. It is possible to zoom in on the high-resolution version of this figure to appreciate the interactions between the different structures. Available online: https://zenodo.org/record/6198513 (accessed on 30 November 2021).

To investigate the regeneration of peripheral nerves within nerve conduits, rat median nerves were injured and 8 mm nerve gaps were repaired with a 10 mm chitosan conduit. Regenerating nerves within the conduits were collected 7, 14, 21, and 28 days after an injury and analyzed using immunofluorescence (Figure 1 and Figure 2). For each time point, one section every millimeter from the proximal to the distal stump was stained with antibodies recognizing Schwann cells (S100β), axons (neurofilament/NF), and endothelial cells (Reca1).

At 7 days after the injury, the Schwann cells, endothelial cells, and neurofilaments were limited to the proximal and distal ends of the conduit, corresponding to the regenerating and degenerating stumps, respectively (Figure 2 and Figures S1–S3, where the single labeling is shown). In the middle portion of the conduit, other cell populations, included in a loose extracellular matrix, filled the conduit; indeed, in this region, cell nuclei were present (a representative section for each time point with DAPI labeling is shown in Figure 1B), while no Schwann cells and endothelial cells are detected.

**Figure 3:**
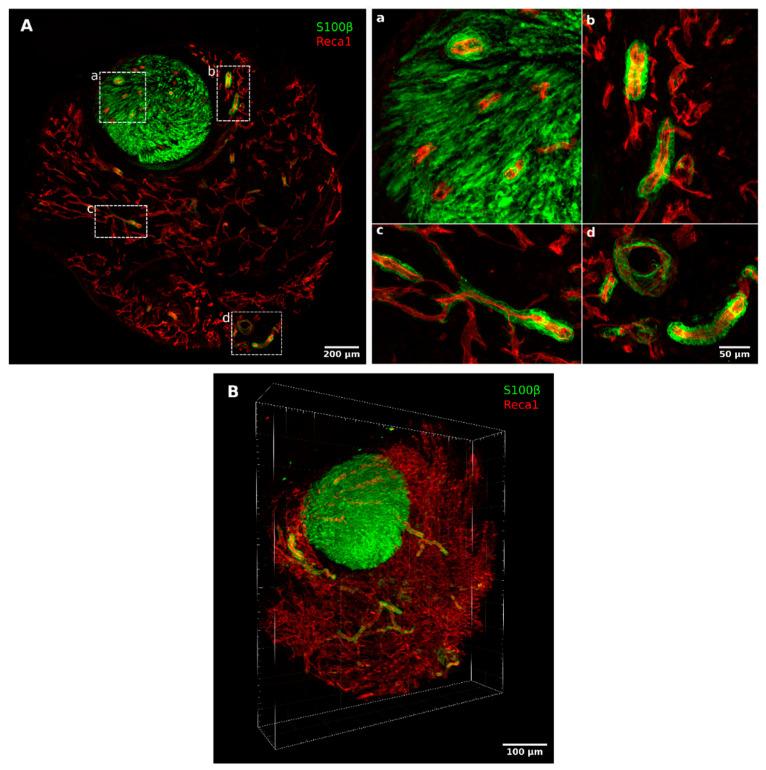
High magnification pictures and 3D reconstruction of a regenerating nerve 7 days after the injury and repair. (**A**) A 50 µm thick section of the distal portion of the conduit double-labeled with Reca1 (red, endothelial cell marker) and S100β (green, Schwann cell marker); (**a**–**d**) inserts: high magnification details of migrating Schwann cells closely associated with endothelial cells. (**B**) Three-dimensional reconstruction of seven consecutive 50 µm thick sections labeled with Reca1 (red, endothelial cell marker) and S100β (green, Schwann cell marker). The 3D reconstruction is available in Video S1 and, at high resolution, here: High resolution 3D reconstruction of regenerating nerve within a chitosan conduit 7 days after injury and repair. Available online: https://zenodo.org/record/5795421 (accessed on 30 November 2021).

One week after the injury, a rich net of newly formed blood vessels surrounded both proximal and distal stumps, and several Schwann cells were closely associated with them (Figure 2 and Figure 3). This association was detectable also within the stumps, as shown in Figure 3A, with a higher magnification of the distal stump.

**Figure 4:**
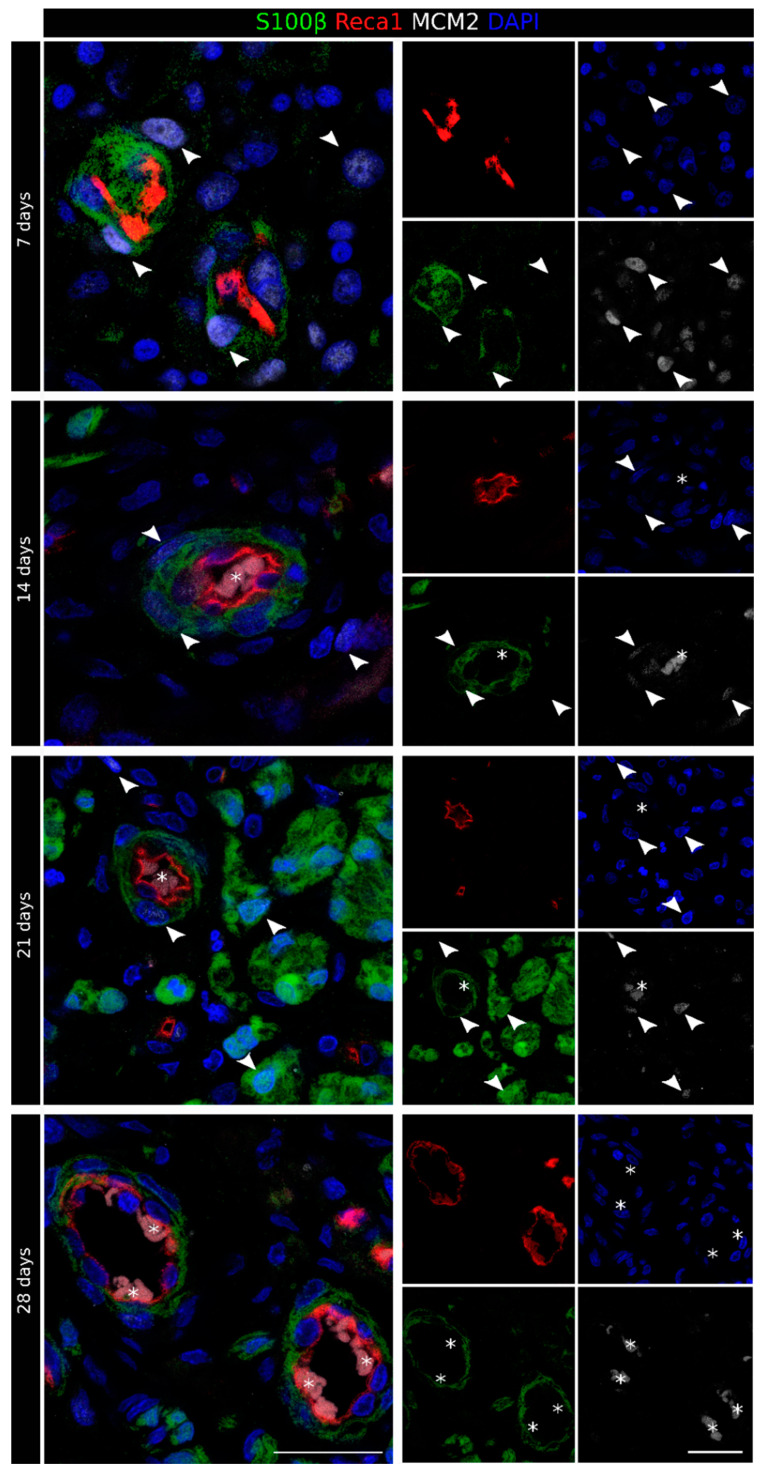
Identification of newly formed Schwann cells in the regenerating nerve. Proliferation marker staining of the regenerating nerve 7, 14, 21, and 28 days after the injury and repair. For each time point, the merged picture is shown on the left, while the four single labeling samples are shown on the right. Sections were labeled with MCM2 (white, proliferation marker), S100β (green, Schwann cell marker), Reca1 (red, endothelial cell marker), and DAPI (blue, nuclear marker). Erythrocyte autofluorescence was detected inside some blood vessels (identified by an asterisk). MCM2-positive Schwann cells are indicated using an arrowhead. Scale bar: 25 µm.

To better highlight this interaction, we performed a 3D reconstruction of seven 50 µm thick consecutive sections of the distal portion, altogether corresponding to 350 µm along the proximo–distal axis (Figure 3B). These images clearly show that, within the conduit, Schwann cells were organized in long chains, closely associated and completely wrapping a subset of the blood vessels, indicating that also in the case of chitosan conduits, these cells used vasculature to migrate from both stumps. The analysis with the proliferation marker minichromosome maintenance 2 (MCM2) [25] showed that 7 days after the injury and repair, several cells were proliferating, among them, Schwann cells migrated on endothelial cells and endothelial cells themselves (Figure 4).

**Figure 5:**
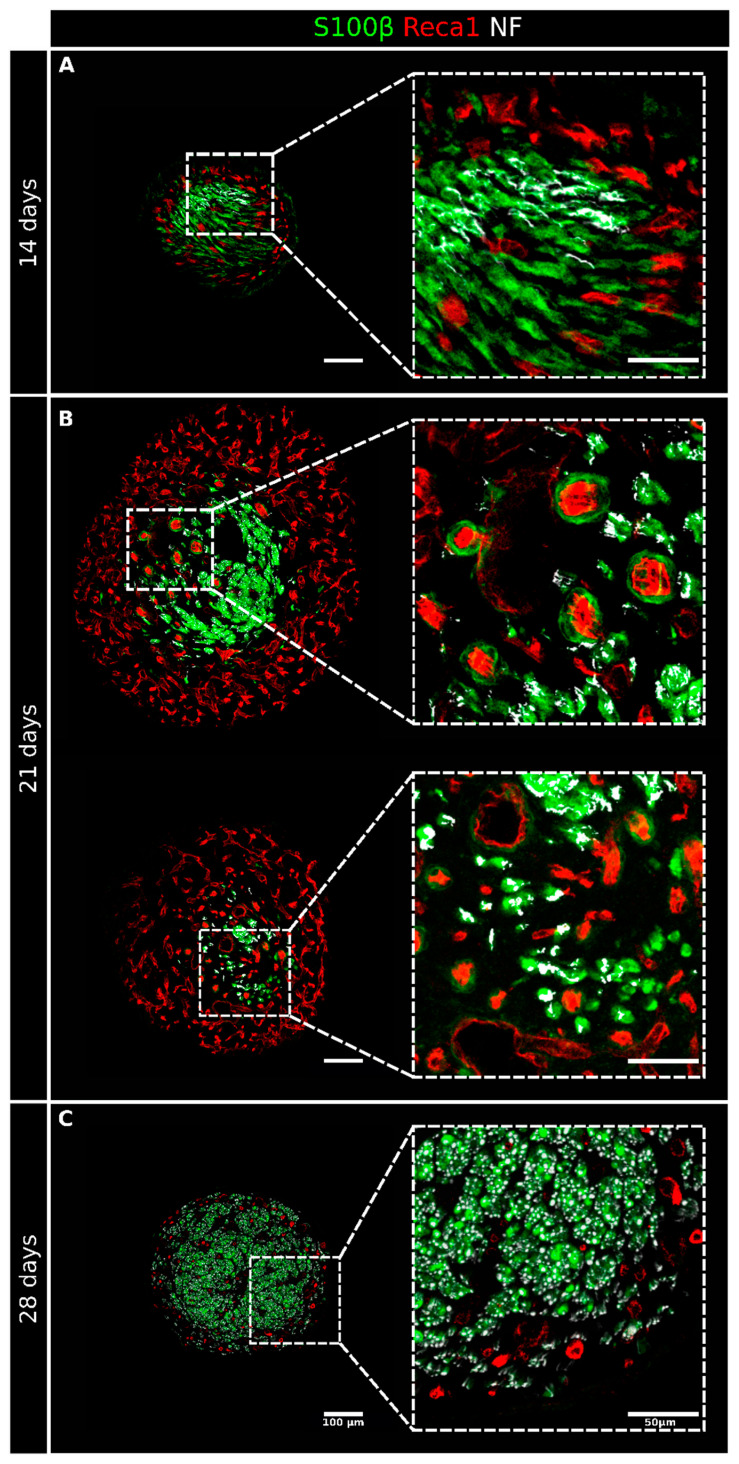
High-magnification details of regenerating nerves 14, 21, and 28 days after the injury and repair. Four sections from Figure 2 are shown here at a higher magnification to better appreciate the interactions between the different structures at 14 (**A**), 21 (**B**), and 28 (**C**) days after the injury and repair. Sections were labeled with Reca1 (red, endothelial cell marker), S100β (green, Schwann cell marker), and Neurofilament/NF (white, axon marker); scale bar: 100 µm, insert scale bar: 50 µm.

At 14 days after the injury, the rich net of blood vessels and Schwann cells had already colonized the entire conduit; regenerating axons, closely associated with Schwann cells, were detectable in most sections (Figure 2 and Figures S1–S3), even if they did not yet reach the distal stump. Migrating Schwann cells associated with blood vessels were still, in part, proliferating (Figure 4). In several sections, it was possible to observe Schwann cells not associated with vessels but instead wrapped with regrowing axons or isolated, preceding the arrival of the regenerating axons (Figure 5A) that use Schwann cell cords as a substrate to cross the nerve gap and reach the distal stump [15].

The diameter of the regenerating nerve within the conduit at 14 days was drastically reduced compared with the diameter at 7 days, while the cellular density was higher due to the formation of the compact structure of the regrowing nerve, as shown in Figure 1 and Figure 2.

At 21 days after the injury (Figure 2 and Figures S1–S3), the diameter of the tissue inside the conduit was considerably increased, mainly because of a thick, vascularized layer surrounding the central core of the regenerating nerve, where several Schwann cells, axons, but also blood vessels were detected. Of note, in the central core (at higher magnification in Figure 5B), blood vessels were more polarized than those in the periphery, as can be appreciated by their round shape in the transverse sections. These longitudinally oriented blood vessels were mostly surrounded by Schwann cells, thus suggesting that these cells were still migrating inside the conduit. The immunofluorescence analysis of the proliferation marker MCM2 showed a very faint signal in a few Schwann cells migrating on endothelial cells (Figure 4). Regrowing axons were more numerous than at 14 days after the injury and followed a similar trend of progressive reduction at increasing distance from the proximal stump, with some of them already reaching the distal stump at this time point (Figure S1). Schwann cells wrapped these regrowing axons, although some of them were still isolated, preceding the arrival of the axons (Figure 5B); some of these Schwann cells were also positive to the proliferation marker (Figure 4).

At 28 days after the injury, the regenerating tissue was slightly thinner than at 21 days, but the nerve core was increased and now occupied the entire sections inside the conduit. A blood vessel crown was still visible only around the proximal and the distal stumps, while the vascularization in the middle portion of the conduit was strongly reduced (Figure 2 and Figure S3). Blood vessels were mainly longitudinally oriented and strongly intermingled with growing axons that, strongly associated with Schwann cells (Figure 5C) at this time point, were abundant and reached the distal stump. In this phase, the association between Schwann cells and endothelial cells was detectable only in the farther sections of the distal stump (Figure 2). The immunofluorescence analysis of the proliferation marker MCM2 suggested that, at this time point, these migrating cells did not proliferate more (Figure 4).

### 2.2. VEGF-A Is Highly Expressed inside the Chitosan Conduit 7 Days after the Nerve Injury and Repair

**Figure 6:**
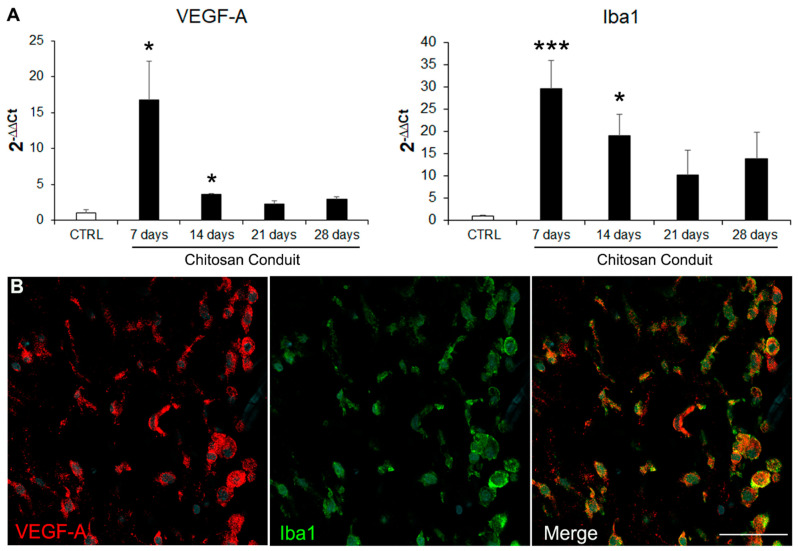
VEGF-A and Iba1 expression. (**A**) Relative quantification (2^−ΔΔCt^) of *VEGF-A* and *Iba1* (macrophage marker) were evaluated using qRT-PCR. Data in the graphs are expressed as mean + standard deviation (*n* = 3 for each group). All data were calibrated to the value of the control uninjured nerves. Asterisks (*) denote significant differences between the uninjured control nerves (CTRL) and the other experimental groups (* *p* < 0.05 and *** *p* < 0.001). (**B**) Immunofluorescence staining of regenerating nerve 7 days after the injury and repair, stained with VEGF-A (red), Iba1 (green, macrophage marker), and DAPI (cyan, nuclear marker). Scale bar: 50 µm.

Since VEGF-A is known to play a key role in angiogenesis and nerve bridge formation [6], we investigated its expression within the chitosan conduit using quantitative expression analysis on samples obtained 7, 14, 21, and 28 days after the injury and repair. The data showed that 7 and 14 days after the repair, *VEGF-A* was significantly more highly expressed in the chitosan conduit than in uninjured nerves, while at 21 and 28 days, its expression level returned to the control values (Figure 6A). Since macrophages were shown to be responsible for detecting hypoxia and subsequently releasing VEGF-A in the nerve bridge model [6], we quantified the expression of the macrophage marker *ionized calcium-binding adapter molecule 1* (*Iba1*, also known as *allograft inflammatory factor 1*, *AIF-1*) within the chitosan conduit at different experimental time points, showing that it was more highly expressed than in uninjured nerves 7 and 14 days after the injury and repair, thus suggesting that VEGF-A may be expressed by macrophages also in the conduit. Accordingly, Iba1 immunostaining confirmed the high presence of macrophages, which were also VEGF-A positive at 7 days after the repair (Figure 6B).

## 3. Discussion

The ability of endothelial cells to provide a track for different cell types was demonstrated in the central nervous system (CNS), where blood vessels were shown to guide neurons [26] and oligodendrocyte precursors [27] during development, neurons in the post-stroke brain [28], and Schwann cells after exogenous transplantation in the demyelinated spinal cord [29].

In the PNS, after a nerve injury, endothelial cells were shown to provide a track for Schwann cell migration across a 2–3 mm nerve bridge, the tissue spontaneously forming between two nerve stumps, which has been extensively studied and characterized by several researchers, showing the role played by different cell populations in nerve regeneration, including fibroblasts, macrophages, Schwann cells, and endothelial cells, as recently reviewed [15,30].

In contrast, the role of endothelial cells in Schwann cell migration within conduits used for the repair of longer gaps was only hypothesized [15] but, as far as we know, not yet demonstrated. As a matter of fact, the environment within a conduit used to repair an 8 mm peripheral nerve gap is different from the environment of the nerve bridge: the first is protected and enclosed by the conduit walls, which somehow could reduce the migration of cells from the surrounding environment, and the migration path is longer. Moreover, the gap nerve repair with a conduit needs microsurgery, while nerve bridge formation is a spontaneous event. The colonization of silicon conduits by different cell populations was described both qualitatively and quantitatively by several authors through light and electron microscopy analysis, but the relationship between Schwann cells and endothelial cells has not been described [20,21,22,23,24].

A time course analysis of nerve regeneration within a chitosan conduit was performed in this study in order to investigate whether blood vessels in the conduit might play the same role as that in the nerve bridge. A chitosan conduit was used because it possesses many of the characteristics that an ideal nerve guide might own, among which, being reabsorbable [31]. Chitosan is widely used to support and promote nerve regeneration and its effectiveness was confirmed by different research groups, including ours [32,33,34,35,36]. Indeed, in previous studies, we analyzed short-term (7, 14, 28 days) and long-term (12 weeks) regeneration of injured median nerves repaired with the same chitosan guide used in this study (Reaxon Nerve Guide, Medovent Gmbh, Mainz, Germany) but using different techniques: morphological and morphometrical analyses, gene expression analysis, and functional assays [32]. This chitosan guide is also already used in clinical practice [37].

A detailed analysis of the regeneration within silicon tubes carried out using electron microscopy showed the presence of blood vessels until the third week after the injury and repair [24]. In a similar way, we previously observed a high number of blood vessels within a chitosan conduit 7 days after the injury and repair [11]. In the current study, using immunofluorescence analysis, we showed that the regenerating tissue maintained an elevated number of blood vessels, not only at 7, 14, and 21 days but also at 28 days after the repair, unlike what was previously described in the silicone tube in which, at 28 days, endothelial cells were not more detectable [24].

Our results showed gradual colonization of the conduit by endothelial and Schwann cells. In fact, 7 days after the repair, endothelial and Schwann cells were detectable only at the extremities, while the major component in the middle of the conduit was represented by the extracellular matrix and other cell types, including macrophages.

At this time point, VEGF-A and Iba1 were highly expressed within the nerve conduit, and their co-localization was observed, thus suggesting that macrophages released VEGF-A under hypoxic conditions, as previously shown by Cattin and colleagues in the nerve bridge [6]. As expected, in the early colonizing steps corresponding to the first few weeks, many cell types, including Schwann cells and endothelial cells, were positive for the proliferative marker MCM2, thus suggesting that they were newly formed cells. Indeed, MCM2 labels all proliferative cells during the active phases of the cell cycle and disappears when cells are quiescent [25].

This might explain the high presence of blood vessels since VEGF-A is a potent pro-angiogenetic factor [38]. The rich vascularization at 7 days was evident in the 3D reconstruction analysis. Interestingly, this analysis shows that Schwann cells detected outside the stumps are all associated with endothelial cells, thus suggesting that they use blood vessels as a track for migration within the conduit, like in the nerve bridge [6].

Fourteen days after the repair, conduit colonization proceeded; indeed, endothelial and Schwann cells were detected in all sections. At this time point, it was more evident that blood vessels were involved in Schwann cell migration and worked like a guide. Moreover, several Schwann cells were observed far from blood vessels, waiting for the axons to reach them [15].

Twenty-one days after the repair, vascularization increased compared with at 14 days, and a rich blood vessel net surrounded the central core of the regenerating tissue. The abundance of vessels at this time point should have more of a nourishment and oxygen supply function. Indeed, a well-represented vasculature is necessary to support the increased metabolic request for Schwann cell proliferation, myelination, and axonal extension [39]. Nevertheless, the guide function of blood vessels is still maintained, but only a few Schwann cells were found to be associated with polarized vessels. The effectiveness in supporting nerve regeneration of longitudinally oriented blood vessels was previously hypothesized [40] but was attributed to the fibronectin matrix, which composed the material used as a conduit to bridge a sciatic nerve gap. In this study, we observed that vessels were longitudinally oriented along the hollow chitosan conduit, therefore allowing Schwann cells to migrate in the right direction to reach the opposite stump and suggesting that this mechanism could be independent of the nerve guide type used. Most of the vessels found at 28 days after the injury and repair were polarized, while Schwann cells were no longer associated with them, as they had stopped migrating and had been achieved by axons. The association between Schwann cells and endothelial cells was maintained only in the last section, where some Schwann cells were still migrating.

It was known that blood vessels supply blood, oxygen, and other nutrients, playing a key role in supporting the peripheral nerve function in physiological conditions and nerve regeneration in pathological conditions [41]. Our results showed that blood vessels not only sustained cell survival but also provided a path for Schwann cell migration, thus suggesting that promoting vascularization might be a good strategy to support and enhance nerve regeneration when angiogenesis is impaired, such as in severe nerve injuries characterized by a long gap, or in elderly patients [42,43].

## 4. Materials and Methods

### 4.1. In Vivo Surgical Procedure

Twenty-three adult female Wistar rats (ENVIGO, Milan, Italy), weighing about 200–250 g, were used. The animal welfare, housing conditions, and surgical procedures were previously detailed [11,32]. All procedures agreed with the National Institutes of Health guidelines, the Italian Law for Care and Use of Experimental Animals (DL26/14), and the European Communities Council Directive (2010/63/EU). Moreover, all procedures were approved by the Bioethical Committee of the University of Torino and the Italian Ministry of Health. Approval code 864/2016-PR (14/09/2016).

Rat median nerves were exposed and transected to establish an 8 mm nerve gap, which was immediately repaired using a chitosan conduit (Reaxon^®^ Nerve Guides Medovent GmbH, Mainz, Germany). A 10 mm long chitosan tube was used to cross the nerve defect by inserting 1 mm of each nerve ending inside the conduit.

Regenerated nerves were withdrawn 7, 14, 21, and 28 days after the repair for biomolecular (*n* = 3, for each time point analyzed) and morphological (*n* = 2, for each time point analyzed) analysis; then, the animals were sacrificed using an anesthetic overdose.

Control nerves for biomolecular analysis were healthy median nerves obtained from 3 uninjured animals.

### 4.2. Immunofluorescence Analysis

Regenerated nerves were collected at 7, 14, 21, and 28 days after the surgery for immunofluorescence analysis (IF). In order to harvest the samples, chitosan conduits were carefully removed, leaving intact the content to minimize the loss of tissue grown inside. Collected nerves were fixed in 4% paraformaldehyde (Electron Microscopy Sciences, Hatfield, PA, USA) in phosphate-buffered saline (PBS, Sigma-Aldrich, Merck, Darmstadt, Germany) for 3 h at room temperature (RT).

In order to cut the nerves into 50 µm thick cross-sections, a cryo-embedding procedure was performed, as described below. First, samples were cryo-protected through passages in solutions with exponentially increasing concentrations of sucrose (Sigma-Aldrich, Merck, Darmstadt, Germany): 7.5% sucrose for 30 min, 15% sucrose for 1 h, and 30% sucrose overnight in PBS at RT. Next, samples were maintained in 30% sucrose–50% optimal cutting temperature medium (OCT, Electron Microscopy Sciences, Hatfield, PA, USA) for 30 min at RT and then embedded in 100% OCT.

In the last passage, specimens were slowly frozen with isopentane (Sigma-Aldrich, Merck, Darmstadt, Germany) thermalized at −70 °C in dry ice and stored at −80 °C. Every sample was entirely cut into 50 µm thick sections with a cryostat (Leica Microsystem S.P.A CM 1900) and around 200 sections were obtained from each sample.

Before IF, floating selected sections were washed 3 times with PBS at RT and then permeabilized and saturated in 2% Triton X-100 (Sigma-Aldrich, Merck, Darmstadt, Germany) and 10% normal donkey serum (NDS, Jackson ImmunoResearch, Philadelphia, USA) in PBS for 4 h at RT in agitation.

Samples were incubated with different combinations of primary antibodies as reported in Table 1. The primary antibodies were diluted in PBS containing 1% NDS and 2% Triton X-100 and the incubation was performed at 4 °C for 48 h with agitation.

Before the secondary antibody incubation, the samples were washed 3 times with PBS at RT. The secondary antibodies (Table 1) were diluted in PBS containing 1% NDS and 0.02% Triton X-100, and the incubation was performed overnight at 4 °C with agitation. Finally, a 15 min incubation in DAPI (ThermoFisher, Waltham, MA, USA) at RT with agitation was performed. After three washes in PBS, sections were placed on a glass slide pre-treated with 1% gelatine (Sigma-Aldrich, Merck, Darmstadt, Germany) and cover-slipped with mounting medium Mowiol (Sigma-Aldrich, Merck, Darmstadt, Germany).

After drying, samples were analyzed using a Leica SP5 confocal microscope (Leica Microsystems, Wetzlar, Germany).

### 4.3. Three-Dimensional Reconstructions

Three-dimensional reconstructions were performed as described previously [44,45]. Briefly, seven consecutive 50 µm thick serial sections were acquired using a Leica SP5 confocal microscope (Leica Microsystems, Wetzlar, Germany) equipped with a 20× objective (HC PL FLUOTAR 20× NA = 0.5) with a voxel size of 0.75 µm × 0.75 µm × 5 µm. By dividing the total number of planes by the 350 µm total depth, we calculated a shrinking along the z-axis of 65% and corrected the voxel size accordingly to 0.75 µm × 0.75 µm × 14 µm. Confocal stacks tiles of each section were stitched with the Grid/Collection stitching plugin in Fiji [46] and the resulting stacks were aligned in TrakEM2 [47]. The reconstructed volume of 1870 (width) × 2115 (height) × 350 (depth) µm was then visualized with Imaris software (Imaris 9.7.2) to produce the representative image (Figure 3B) and the Supplementary Video S1 available at high resolution, here: High resolution 3D reconstruction of regenerating nerve within a chitosan conduit 7 days after injury and repair. Available online: https://zenodo.org/record/5795421 (accessed on 30 November 2021).

### 4.4. RNA Extraction, cDNA Preparation, and Quantitative Real-Time PCR (qRT-PCR) Analysis

Total RNA isolation, retrotranscription, and qRT-PCR were performed using 0.75 µg/sample for retrotranscription, as previously described [48]. For the qRT-PCR analysis, technical and biological triplicates were performed and the obtained data for relative quantification were analyzed using the “Livak 2^−ΔΔCt^ method” [49], as previously described [11]. The average of uninjured nerves was used as the calibrator for the relative quantification; for data normalization, the geometric average of *RICTOR (RPTOR Independent Companion of MTOR, Complex 2)* and *ANKRD27 (Ankyrin repeat domain 27)* threshold cycles were used, as previously described [50]; primer sequences are available in the same paper. Primer sequences for *VEGF-A* and *Iba1* were as follows. *VEGF-A*: forward 5′-ACCATGAACTTTCTGCTCTCTTGGG-3′, reverse 5′-CTTCATGGGCTTTCTGCTCCCC-3′; *Iba1*: forward 5′-GATGGGATCAACAAGCACTTCCTCG-3′, reverse 5′-CTCCATGTACTTCGTCTTGAAGGCC-3′.

### 4.5. Statistical Analysis

Statistical analysis was executed using IBM SPSS Statistics 25 software 27. qRT-PCR data were expressed as mean + standard deviation. The presence of a normal distribution was tested for all data (Levene’s and Mauchly’s tests) to select the appropriate statistical test. One-way analysis of variance (ANOVA) or the Kruskal–Wallis test was adopted for parametric or non-parametric data, respectively, with Bonferroni’s correction to highlight differences between the control and experimental groups.

## Figures and Tables

**Table 1:** Primary and secondary antibodies for immunofluorescence analysis (Abcam, Cambridge, UK; Bio-Rad, Hercules, CA, USA; FUJIFILM Wako Chemicals Europe GmbH, Neuss, Germany; Jackson ImmunoResearch, Philadelphia, PA, USA; Santa Cruz Biotechnology, Heidelberg, Germany; Sigma-Aldrich, Merck, Darmstadt, Germany).

Antibodies for Immunofluorescence Analysis				
Primary Antibodies				
	Code	Dilution	Host	Source
Neurofilament/NF	Ab4680	1:10,000	Chicken	Abcam
S100β	HPA015768	1:200	Rabbit	Sigma-Merck
Reca1	MCA970R	1:500	Mouse	Bio-Rad (AbD Serotec)
Iba1	019-19741	1:1000	Rabbit	FUJIFILM Wako Chemicals U.S.A. Corporation
VEGF-A	ab1316	1:200	Mouse	Abcam
MCM2	sc-9839	1:500	Goat	Santa Cruz Biotechnology
Secondary Antibodies				
	Code	Dilution	Host	Source
AlexaFluor 488 Anti-rabbit	711-545-152	1:400	Donkey	Jackson ImmunoResearch
AlexaFluor 488 Anti-goat	705-545-147	1:400	Donkey	Jackson ImmunoResearch
Cy3 Anti-Mouse	715-165-151	1:800	Donkey	Jackson ImmunoResearch
Cy3 Anti-rabbit	711-165-152	1:800	Donkey	Jackson ImmunoResearch
AlexaFluor 647 Anti-chicken	703-605-155	1:800	Donkey	Jackson ImmunoResearch
AlexaFluor 647 Anti-mouse	715-605-151	1:800	Donkey	Jackson ImmunoResearch

## Data Availability

The row data presented in this study are available on request from the corresponding author.

## Supplementary Materials

The following supporting information can be downloaded at: https://www.mdpi.com/article/10.3390/ijms23042254/s1.
