# Supplementary material for: Blood Vessels: The Pathway Used by Schwann Cells to Colonize Nerve Conduits
Source: Int J Mol Sci. 2022 Feb 18;23(4):2254. doi: 10.3390/ijms23042254 (PMC8879195; doi:10.3390/ijms23042254)
Supplement: Supplementary file 1 [file ijms-23-02254-s001.zip › Supplementary materials.pdf]

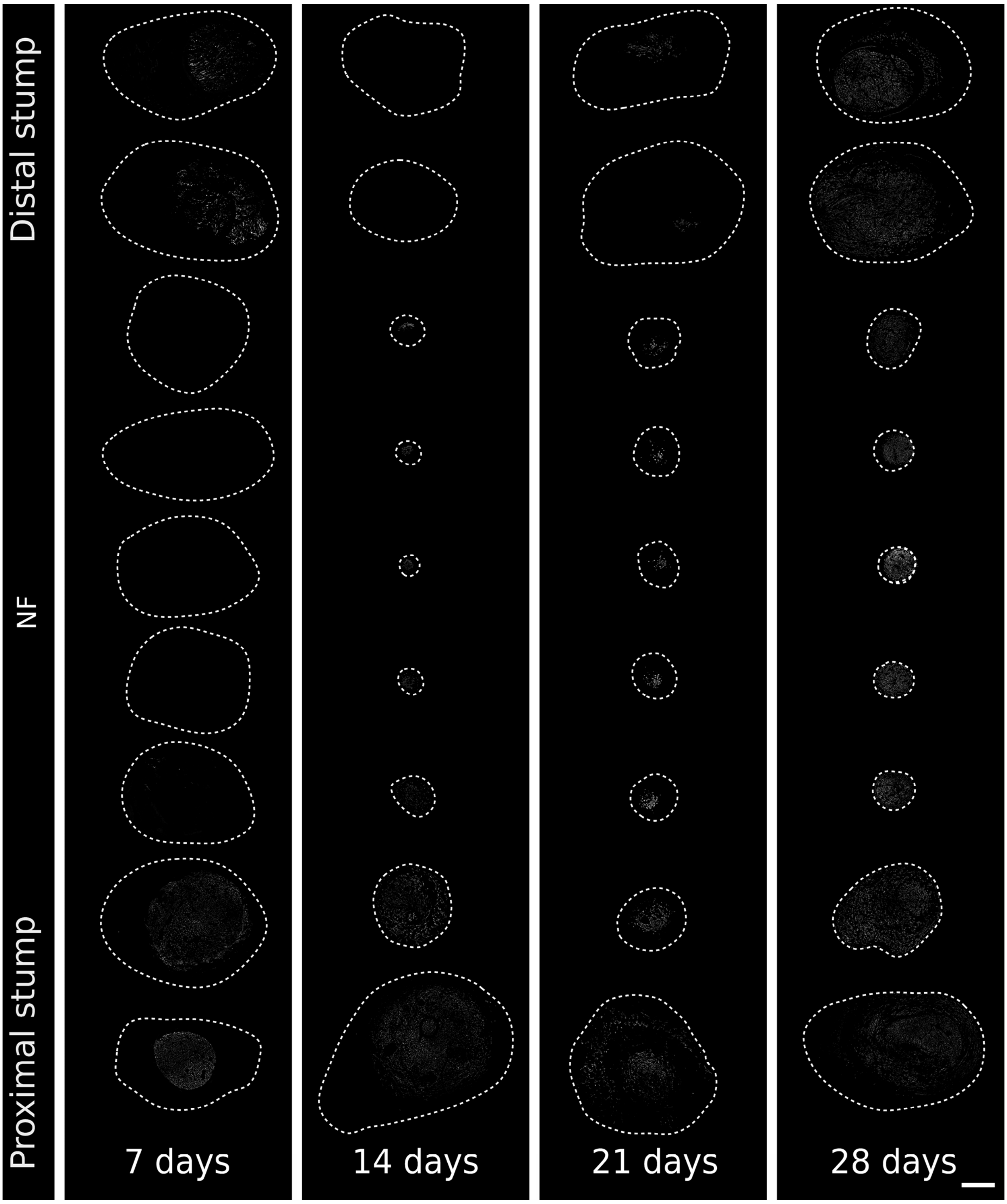

Figure S1: Immunofluorescence staining of regenerating nerves 7, 14, 21, and 28 days after the injury and repair. One section every millimeter was labeled with Neurofilament/NF (white) to follow the axon progression, as shown in Figure 2. The dotted line delimits the region containing cell nuclei identified with DAPI (data not shown).

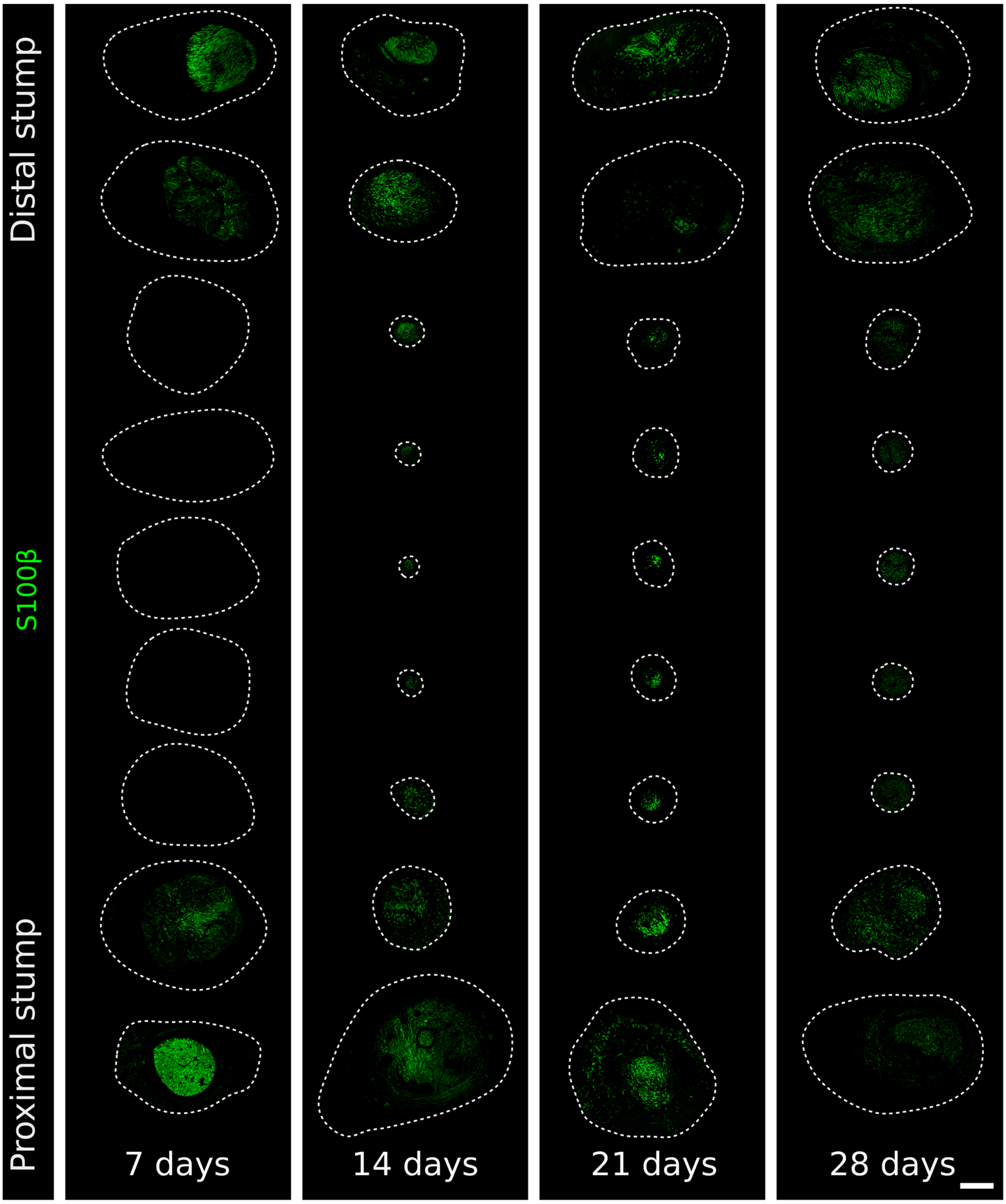

Figure S2: Immunofluorescence staining of regenerating nerves 7, 14, 21, and 28 days after the injury and repair. One section every millimeter was labeled with S100β (green) to follow the Schwann cell progression, as shown in Figure 2. The dotted line delimits the region containing cell nuclei identified with DAPI (data not shown).

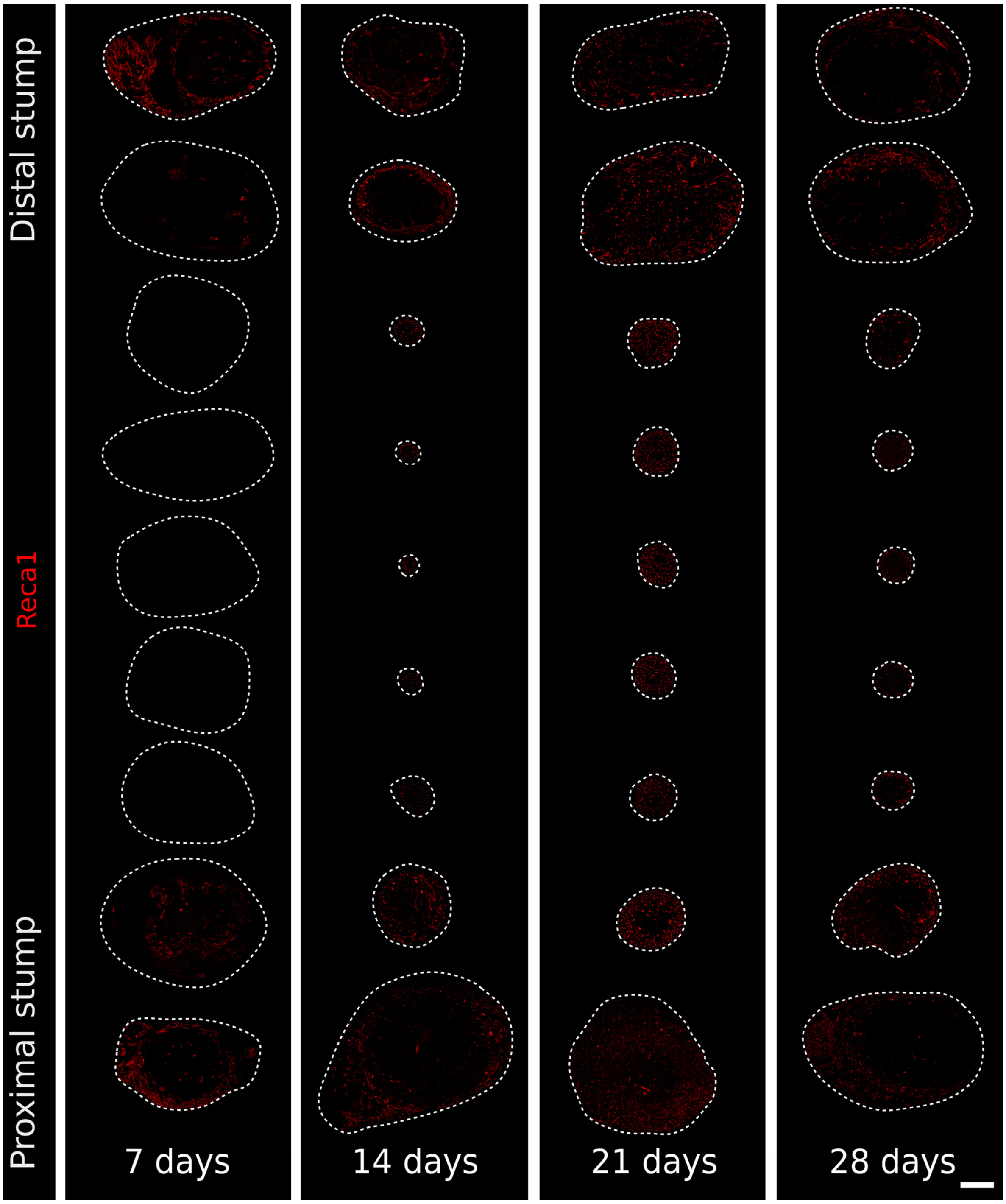

Figure S3: Immunofluorescence staining of regenerating nerves 7, 14, 21, and 28 days after the injury and repair. One section every millimeter was labeled with Reca1 (red) to follow the blood vessel progression, as shown in Figure 2. The dotted line delimits the region containing cell nuclei identified with DAPI (data not shown).

Video S1: Three-dimensional reconstruction of 7 consecutive 50 μm thick sections labeled with Reca1 (red, endothelial cell marker) and S100β (green, Schwann cell marker). A high-resolution 3D reconstruction video is also available here: High resolution 3D reconstruction of regenerating nerve within a chitosan conduit 7 days after injury and repair. Available online: https://zenodo.org/record/5795421 (accessed on 15 February 2022).
